# Gender focused training and knowledge enhances the adoption of climate resilient seeds

**DOI:** 10.1016/j.techsoc.2020.101388

**Published:** 2020-11

**Authors:** Manzoor H. Dar, Showkat A. Waza, Swati Nayak, Ritadhi Chakravorty, Najam W. Zaidi, Mosharaf Hossain

**Affiliations:** a AFC India Limited (Formerly Agricultural Finance Corporation Ltd), Kirti Nagar, New Delhi, India; b MCRS Sagam (Khudwani), SKUAST of Kashmir, J&K, India; c International Rice Research Institute (IRRI-India), NASC Complex, New Delhi, India; d Reserve Bank of India, New Delhi, India

**Keywords:** Quality seed production (QSP) trainings, Climate resilient or stress tolerant rice varieties (STRV), Informational intervention, Adoption, Gender

## Abstract

Adoption of any agricultural technology depends upon the way in which farmers are being informed about its benefits. Educational status, caste, gender and other social issues also play a significant role in the adoption process. To evaluate the impact of trainings on quality seed production, access to the climate resilient rice seeds, availability of information about seed sources and use of IRRI super bags, a randomized experimental research was carried out over a period of two years across five different states of India. The baseline and a follow-up survey was conducted to capture the farming practices followed by during wet seasons of 2016 and 2017, respectively. The impact of trainings, seed use, information given and agro-based goods was evaluated by comparing the adoption behaviour of treatment and control farmers. There was an increase (28.8%) in the practice of using salt solution to clean seeds primarily due to the impact of quality seed production (QSP) trainings. Female farmers responded more than the male farmers as number of women adopting the practice was higher than men. The impact of the trainings on farmers' knowledge and adoption of climate resilient/stress tolerant rice varieties (STRV) was also more pronounced on females than on male farmers. Farmers’ access to seeds substantially enhanced the adoption and reusability irrespective of the gender. Similarly, the information delivered to the farmers was quite economical in enhancing the awareness and adoption of climate resilient rice, but the effect was predominantly driven by female farmers. Female farmers performed relatively better with respect to the storing the new seeds in IRRI super bags. Thus, incentivising farmers in general and female farmers in particular can serve as a potential means to adopt agricultural technologies that have potential to boost rural economy and enhance the food security. The results are being supported by a rigorous empirical analysis.

## Highlights

• The study provides with evidence that an increase in seed cleaning practice is primarily due to the impact of trainings.
• The information intervention is economical that enhances the adoption of new seeds and is mostly driven by female farmers.
• Incentivising female farmers can serve as a potential means to boost rural economy and enhance the food security.

## 1 Introduction

Agricultural productivity has a direct and major role to play in addressing the concern of food insecurity. In a country like India where 70% of population resides in rural areas with agriculture as a major source of livelihood, agricultural productivity remains a dominant determinant for overall welfare of the nation [1]. Green revolution driven enhancement in agricultural productivity has significantly affected food grain production in India that has reached to the level of 206 mt from 103 mt, between 1970 and 2000 [2]. Green revolution has established with evidence that the adoption of high yielding varieties (HYVs) of major food crops like rice can play a key role in increasing farm income through the productivity growth [3]. Although, the massive popularization of modern HYVs of rice developed during and after green revolution has directly contributed to hunger and poverty reduction measures, the yield potential of these varieties can't be increased beyond the supporting capacity of soil, water and other resources. Moreover, these HYVs have mainly targeted the better-endowed areas under assured irrigation. The benefits of green revolutionary HYVs of rice have remained elusive for rainfed ecosystems, particularly those affected by abiotic stresses especially flooding and drought [4]. Strategies focusing to enhance the rice productivity under abiotic stress prone ecologies will be helpful in improving the overall productivity and thereby enhancing the food security [5].

The frequency, intensity and duration of abiotic stresses is expected to rise due to the progressive effects of climate change, ensuing resilient yields under these conditions to be increasingly important [6]. Making rice production more resilient to stresses and occasional weather shocks is an important component of overall food security. Rapid progress has been made in developing the climate resilient or stress tolerant rice varieties (STRV) and continuous attempts are being made to disseminate these varieties to farmers growing rice under unfavorable ecologies [5,7]. While this remains a concern, the national systems of Indian agriculture has made substantial efforts to introduce and promote the adoption of climate resilient rice varieties to stabilize and improve the productivity further [8]. The most important challenge we face today is the poor adoption and low varietal replacement rates for these stress tolerant varieties [9].

The adoption per se is a dynamic process and in case of improved varieties, it may take several years to reach the maximum level [10]. Within a dynamic process of adoption, often in developing economies, farmers are traditionally disadvantaged in terms of economies of scale (small landholdings), local access to technology and inputs, information and knowledge, enabling infrastructure, credit facilities, and socio-cultural barriers [11]. While some of these adoption constraints need a long term systemic effort to bring visible change, many are directly relevant to extension approaches followed at national level. Moreover, adoption of any technology depends upon the way in which farmers are being informed about its benefits [12]. Trainings imparted to farmers and provision of incentives can help in a better way than mere information [13]. Educational status, caste, gender and other social issues can also play a significant role in an adoption process [11,14,15]. In developing economies like India, with existing socio-cultural barriers besides historical disadvantages for women and differentiated gender based needs in agriculture, the intensity and effect of adoption constraints could further be layered on the basis of gender of adopter [16]. Often it is believed that female farmers tend to adopt improved technologies at a rate different than that of male farmers [17]. It is imperative to identify and develop the concrete evidences of gendered impact on technology, information and knowledge interventions in the adoption process so as to develop the strategies for addressing the constraints under real field conditions [18].

While the adoption puzzle remains pertinent and the constraints to adoption remain many, a randomized control trial was carried out over a period of two years across five different states of India. The areas targeted represent the abiotic stress prone ecologies mainly affected by drought. The main objective of this study was to measure the impact of trainings (regarding quality seed production), local access to technology (seeds of climate resilient rice varieties), access to information (regarding local source of the seeds) and agro-based goods (IRRI super bags) on adoption and knowledge of both male and female farmers. While comparing the treatment and control farmers, this study also reveals the dissemination rate of climate resilient seeds in addition to its adoption.

## 2 Materials and methods

### 2.1 Experimental site and design

The areas targeted represent the abiotic stress prone ecologies mainly affected by drought. The seeds of a drought tolerant rice variety, Sahbhagi Dhan were distributed amongst the selected farmers. This is a short duration variety that has a potential to withstand the drought and therefore stabilize yields under the rainfed environments. The present study was carried out in 61 villages spread over 34 districts in Eastern Indian states of Bihar, Chhattisgarh, Jharkhand, Odisha and West Bengal. A random sample of 10 male and 10 female farmers was selected from each village, thereby selecting a total of 1220 farmers. The villages were randomly allocated to one of the two groups. The first group (V_1_) comprised of 26 villages where both seeds of drought tolerant variety and quality seed production (QSP) trainings were provided to the farmers. The second group (V_2_) consisting of 35 villages where no such trainings were imparted. However, the distribution of seeds for on-farm demonstrations was carried out in both the groups of villages. In each of the V_1_ villages, the sampled farmers were randomly and equally distributed into two groups. The first group (T_1_) received both seeds and QSP trainings. The second group was a pure control (C_1_) which neither received the seeds nor any training. In the V_2_ villages, the sample farmers were again randomly and equally distributed into two groups. The first group (T_2_) received seeds, while the members of second group (C_2_) represent the control and were only informed about the names of T_2_ farmers residing in their respective villages (Table 1). In each of the V_1_ and V_2_ villages, equal proportion of male and female farmers were allocated to each of the treatment (T_1_/T_2_) and control (C_1_/C_2_) groups.

**Table 1:** Allocation of farmers into treatment and control groups in different villages.

Villages	Farmers allocated
V_1_	T_1_ (Received both STRV seeds and QSP trainings)
	C_1_ (Neither received STRV seeds nor QSP trainings)
V_2_	T_2_ (Received STRV seeds only)
	C_2_ (Received information about T_2_ farmers residing in their villages)

T(T_1_/T_2_) and C(C_1_/C_2_) represent treatment and control farmers, respectively.

The primary selection of farmers and a baseline survey to know about their existing farming practices was conducted before the on-set of wet season in 2016. Subsequently, the distribution of seeds to be sown in the oncoming wet season was carried out amongst the T_1_ and T_2_ farmers. The QSP trainings were imparted to the selected subset (T_1_) of farmers during the wet season of 2016. The information intervention to C_2_ farmers was also accomplished during the same season. During the wet season of 2017, all the four subsets of farmers were again contacted for a follow-up survey to assess the impact of the trainings, seed dissemination and information. The follow-up information was collected from a total of 988 farmers which implies an overall attrition rate of about 20%. In order to appraise the impact of agro-based goods, use of IRRI super bags was used as a benchmark. IRRI super bags are hermetic moisture-proof bags developed by IRRI for seed storage. These bags were distributed to farmers during the QSP trainings. Since the bags are not available in market and were thus available with T_1_ farmers only. During the follow-up survey, the farmers (T_1_) were enquired about their usage of IRRI super bags.

### 2.2 Impact of trainings, seed distribution, information intervention and agro-based goods

The impact of QSP trainings was evaluated by comparing the adoption behaviour of T_1_ and T_2_ groups of farmers during the follow-up survey. The farmers were enquired whether they performed salt cleaning of seeds prior to the preparation of nursery beds during 2017. Cleaning of seeds with salt solution was a key point imparted during the QSP trainings. Salt cleaning is an easy and inexpensive to implement, and was thus used as an index to test the effectiveness of the trainings. Along with the adoption of salt cleaning, the impact of QSP trainings on adoption and sharing of drought tolerant seeds was also discerned. While QSP trainings did not explicitly focus on varietal adoption and sharing, both T_1_ and T_2_ farmers were informed about the benefits of seeds and encouraged to share these with the fellow farmers. They were also informed that the seeds can be re-used over years. The impact of STRV seed distribution and information intervention was also appraised. We classified the sources of knowledge about these seeds into formal and local sources. The former comprised of NGOs that informed the farmers about these climate resilient rice varieties, while the latter consists of local farmers who apprised their fellow farmers about STRV seeds. During the follow-up survey, T_1_ farmers were enquired about the use of IRRI super bags for storing seeds. Physical verification of the bags was also carried out to verify about their present condition and if any seed was stored in the bags.

### 2.3 Differential effects by gender

Each of the treatment and control farmer groups in their individual villages consists of the randomly selected sample of 5 male and 5 female farmers. This provides us with a second stratum of randomization to identify differential effects across the gender. The differential effects by gender was evaluated for the key components such as training imparted, adoption of drought tolerant rice amongst seed recipients and their acceptance amongst non-recipient farmers. The gender based impact of information intervention, differential use of IRRI super bags and sharing of seeds amongst the male and female farmers was also appraised.

### 2.4 Conceptual framework and setting up of hypothesis

The present study evaluates an effectiveness of trainings in enhancing the adoption of practices recommended to farmers. Whether the provision of climate resilient seeds led to their increased adoption and sharing amongst the farmers was also appraised. The allocation of farmers into the treatment and control groups were carried out on random basis, therefore it is expected that higher proportion of farmers would have carried out salt cleaning of seeds in T_1_ compared to C_1_ during the follow-up year. There should be no substantial difference in the share of farmers reported to have undertaken the salt cleaning between T_2_ and C_2_ groups. Any difference can be attributed towards the spill over effect of the seed distribution to the treatment farmers. Similarly, the rate of adoption of climate resilient rice may be higher amongst T_1_ than T_2_ farmers due to positive spill overs from QSP trainings. Moreover, a higher proportion of C_2_ farmers is expected to take up new seeds relative to the C_1_ farmers. Information about climate resilient seeds from local sources should be responsible for this higher share of farmers in C_2_ group. Likewise, a higher proportion of farmers is anticipated to report of using IRRI super bags within the T_1_ group. Besides, a higher share of female farmers would be storing seeds in the IRRI super bags or would have kept IRRI super bags safely for future usage.

### 2.5 Empirical analysis

A rigorous empirical analysis was carried out to verify the descriptive trends revealing an impact of QSP trainings, seed distribution, information intervention and agro-based goods. A fixed effect regression model was used for testing the specification similar to that described by Dar et al. [19].

$$Y_{iv}=\;\alpha_{b}+\;\beta_{1}Treatment_{iv}+\;\beta_{2}Treatment_{iv}Female_{iv}\;+\;\beta_{3}Female_{iv}\;+\;\beta_{4}X_{iv}\;+\;\varepsilon_{iv}$$

In above equation, ‘*i*’ and ‘*v*’ represent the farmers and villages respectively, with individual responses forming a unit of observation. *‘Y’* represents an outcome of interest, which is almost always in the form of binary responses such as performing of salt cleaning, adoption of STRV seeds or usage of IRRI super bags. Block-level fixed effects controlling the characteristics common to all farmers such as geographical region or cultural features specific to a block are expressed in the terms of *‘α’*. Farmer-level covariates are included in the vector *X*. As almost all our outcomes of interest are binary measures, the regressions are estimated using the linear probability model. The average treatment effect is measured as *β*_*1*_ whereas *‘Treatment’* represents the treatment status for an individual which may be providing STRV seeds and QSP training (T_1_) or information intervention (C_2_). *‘Female’* indicates a binary indicator having the value 1 if it's a female and 0 otherwise. Thus, *β*_*2*_ represents the differential effect of QSP training or information intervention on females over males. The sum of *β*_*1*_ and *β*_*2*_ indicates the net impact of QSP training on females.

## 3 Results and discussion

### 3.1 Impact of QSP trainings

**Fig. 1:**
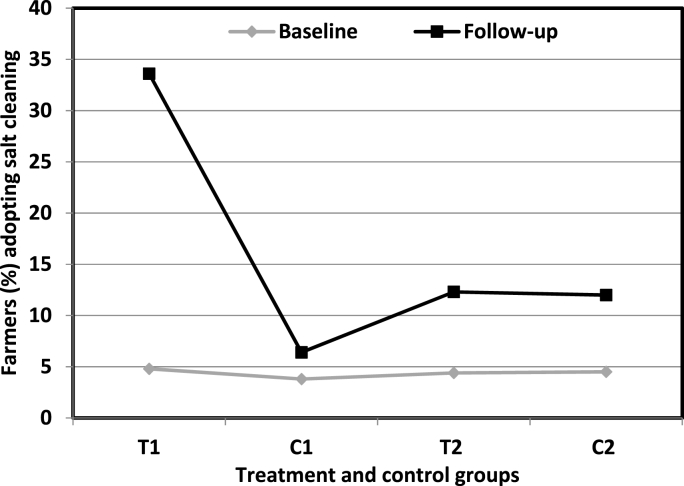
Impact of QSP trainings in terms of the proportion of farmers reported to have carried out salt cleaning during the follow-up survey.

The allocation of farmers into treatment and control groups was carried out on random basis. Therefore, any difference in the share of farmers reported to have carried out salt cleaning across the groups may mainly be attributed to the causal impact of QSP trainings. Fig. 1 presents the impact of QSP trainings in terms of the proportion of farmers reported to have undertaken salt cleaning during the follow-up survey. The impact of QSP trainings was quite encouraging as over a third of farmers who received the trainings (T_1_) reported to have undertaken salt cleaning in the subsequent season. The corresponding share for non-trained (C_1_) farmers was about 6%, which is substantially lower than that of T_1_ (34%) in comparison to 12% of the respondents reported to have undertaken salt cleaning in either of T_2_ or C_2_ groups. This provides the evidence that an increase in salt solution usage to clean seeds during the follow-up year is primarily due to the impact of QSP trainings. Studies suggest that an investment in agricultural trainings is necessary to meet the food requirements of ever increasing population, improving food security in developing economies and enable the farmers to adapt towards changing climatic conditions [20,21]. Moreover, ensuring equal opportunities for men and women in extension trainings can be helpful in increasing food production for the family as whole [22].

**Fig. 2:**
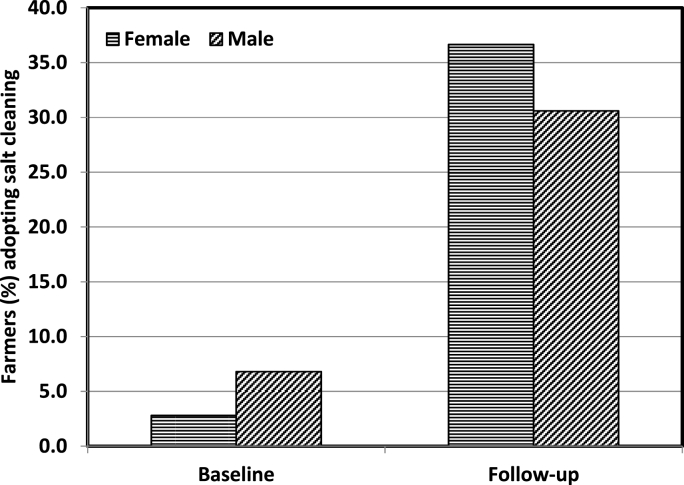
Impact of QSP trainings in terms of the proportion of male and female farmers reported to have undertaken salt cleaning during the follow-up survey (only T_1_ farmers were included).

**Fig. 3:**
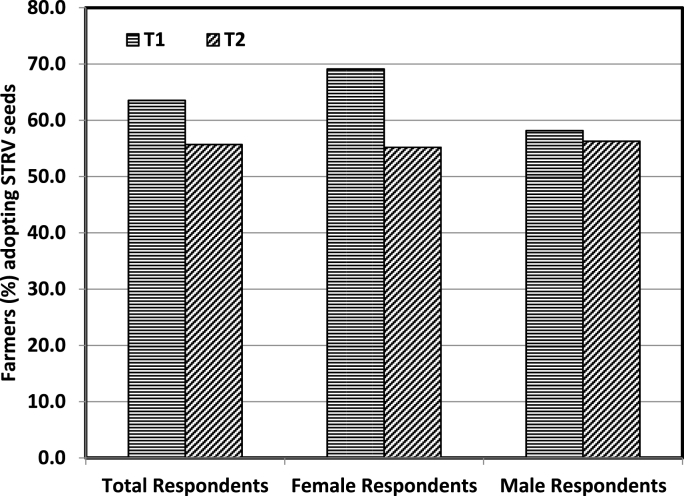
Impact of QSP trainings on adoption and reusability of STRV seeds.

While evaluating the impact of QSP trainings by gender, we restricted our sample to T_1_ and compared across the male and female farmers (Fig. 2). During the baseline, only about 3% of the female farmers reported to have undertaken salt cleaning of seeds, compared to almost 7% of the male farmers. However, a higher share of female farmers (37%) reported to have adopted salt cleaning relative to male farmers (31%) during the follow-up survey. This indicates that the QSP trainings impacted female farmers more than the male farmers. The impact of QSP trainings on adoption and reusability of new seeds was depicted by making the comparison between T_1_ and T_2_ groups (Fig. 3). Although, nearly 64% of the T_1_ farmers planted new seeds during the follow-up year, the corresponding statistic in the T_2_ group was about 56%. The reason may be that the farmers who received QSP trainings (T_1_) might be more favourably inclined towards reusing seeds due to a sense of reciprocity for the time and efforts that trainers have invested for them. Moreover, the difference was being driven by female farmers as nearly 69% of female farmers adopted seeds in T_1_ compared to around 55% in T_2_. For male farmers, there was only a slight difference in adoption rates across the two groups. This also implies that the impact of QSP trainings on adoption and reusability of seeds was more pronounced on females than on male farmers. Although, agricultural trainings have substantially positive consequences across the gender of farmers, the need for women involvement in agricultural training programs is crucial to ensure that the full potential of the trainings is exploited towards enhancing the agricultural productivity [23]. However, studies have revealed that women participation in agricultural trainings is significantly lesser in proportion compared to their partner male farmers [20]. Therefore, the factors that prevent women from participating in training programs should be taken into consideration while planning for any such activities. Extension agencies should also find the ways of empowering women to participate in training programs [24].

**Fig. 4:**
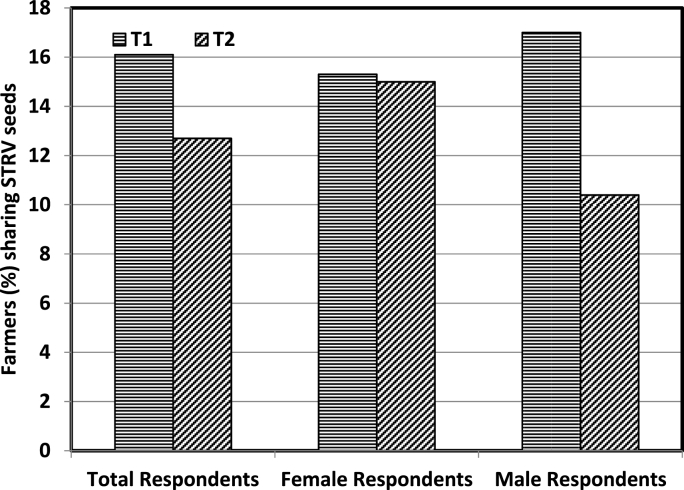
Impact of QSP trainings on sharing of STRV seeds.

Along with the information on reusability of drought tolerant seeds, both T_1_ and T_2_ farmers were encouraged to share these with other fellow farmers in their villages. The impact of QSP trainings on seed sharing was also evaluated through the comparison of T_1_ and T_2_ groups. The levels of sharing during the follow-up were low across the two groups or across the gender (Fig. 4). Thus, while QSP trainings had a large spill over impact on varietal adoption, the corresponding effect on seed sharing was relatively miserable. The most probable reason may be the reluctance of farmers to offer seeds to other fellow farmers despite the norms of mutual assistance and sharing [25]. Moreover, in the present study, the impact of QSP trainings on seed sharing was more on male farmers than on female farmers. Sharing of seed and other resources is a function of social associations and the respect that people have for each other [26]. Farmers who interact with more people have relatively more opportunities of sharing the ideas and resources. Similarly, those who hold leadership positions are more likely to share resources than others [27,28]. Thus, male farmers with more outreach and dominance in decision making are more likely to share seeds with other farmers.

### 3.2 Impact of the distribution of STRV seeds

**Fig. 5:**
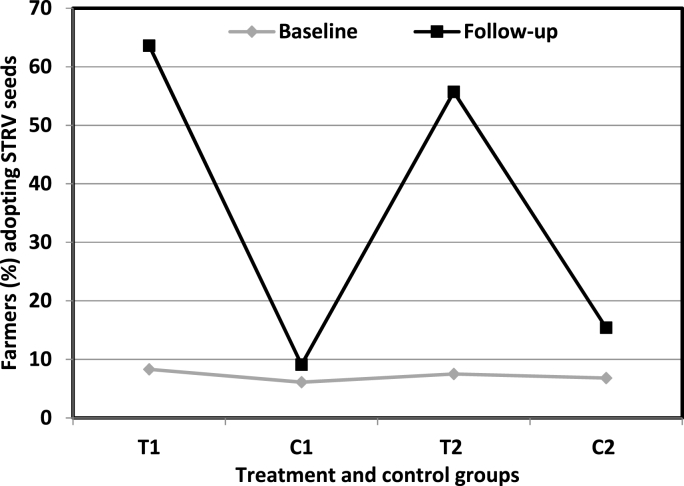
Impact of seed distribution in terms of the share of farmers adopting STRV seeds during the follow-up year.

**Fig. 6:**
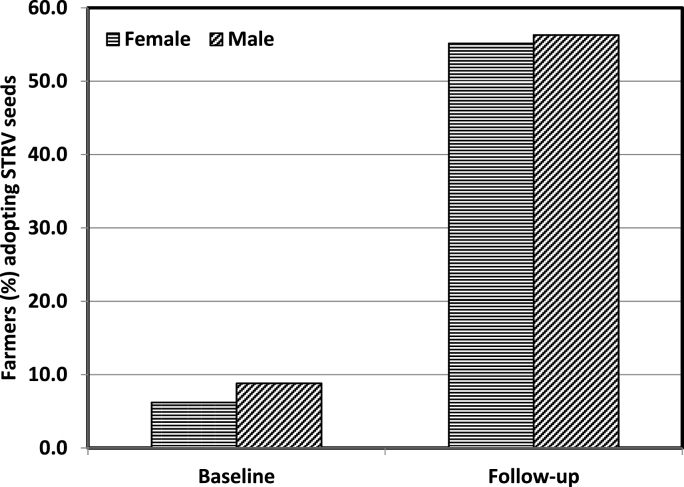
Impact of seed distribution in terms of the share of male and female farmers adopting STRV seeds during the follow-up year (only T_2_ farmers were included).

The main intention behind the seed distribution was to enhance the adoption and reusability of new seeds amongst farmers. Since the allocation of farmers into treatment and control groups was carried out on random basis, any difference in the share of farmers reported to have adopted seeds during the follow-up year may mainly be attributed to the impact of seed distribution. The impact of seed distribution in terms of share of the farmers that reused seeds during the follow-up year is being presented in Fig. 5. A total of 56% of the farmers who had received seeds in T2 group continued for the next season as compared to a relatively higher (64%) in T_1_ group, which may be due to the joint outcome of QSP trainings and seed access. While evaluating the impact of seed distribution by gender, we restricted our sample to T_2_ group and compared across the male and female farmers (Fig. 6). During the baseline, about 6% of the female farmers reported to have planted these seeds, compared to almost 9% of the male farmers. Similarly, slightly lower share of female farmers (55%) reported to have adopted new seeds relative to male farmers (56%) during the follow-up survey. This indicates that gender is not a significant determinant in deciding the adoption of new seeds. Studies have revealed that an access to improved seeds is an integral factor for stimulating technology uptake by the farmers irrespective of their gender [29,30]. However, an impact study by Morris et al. [31] revealed that improved cultivars have been adopted less extensively by women than by men farmers. Nevertheless, improved seed is the primary input for modern agriculture and its distribution has direct impact on agricultural productivity, food security and sustainable economic growth [32,33].

### 3.3 Impact of information intervention

**Fig. 7:**
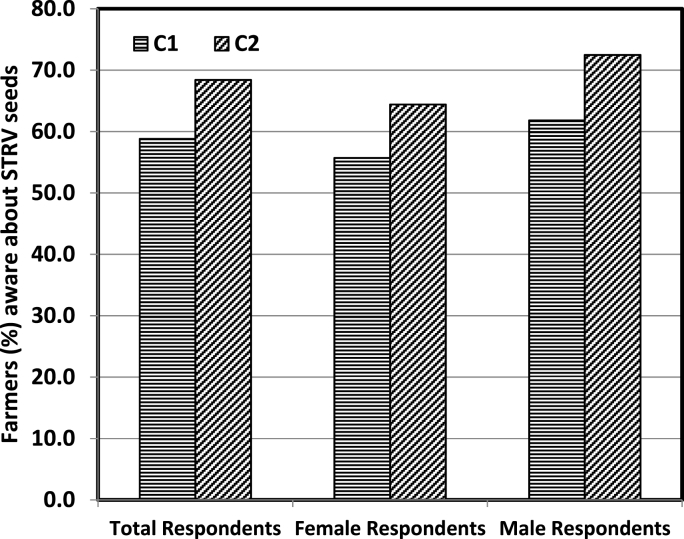
Impact of information intervention on awareness of farmers regarding STRV seeds.

The main objective behind this treatment was to enhance the popularity and adoption of climate resilient rice varieties amongst farmers. During the follow-up survey, nearly 68% of the farmers in C_2_ group responded in an affirmative way regarding their awareness of drought tolerant seeds compared to about 59% of farmers in C_1_ group (Fig. 7). This difference is attributed to the only difference between C1 and C2 groups which is the information about the seeds and seed recipients provided to the C2 farmers. The difference was highly prominent across the male respondents but was also observed amongst female farmers. About 73% of the male farmers in group C_2_ were aware of new seeds compared to 62% in group C_1_. For female farmers, 64% in group C_2_ were aware of these seeds compared to 56% in group C_1_. The informational interventions have the potential to enhance the knowledge and confidence levels of both male and female farmers, thereby improving their decision making abilities [34].

**Fig. 8:**
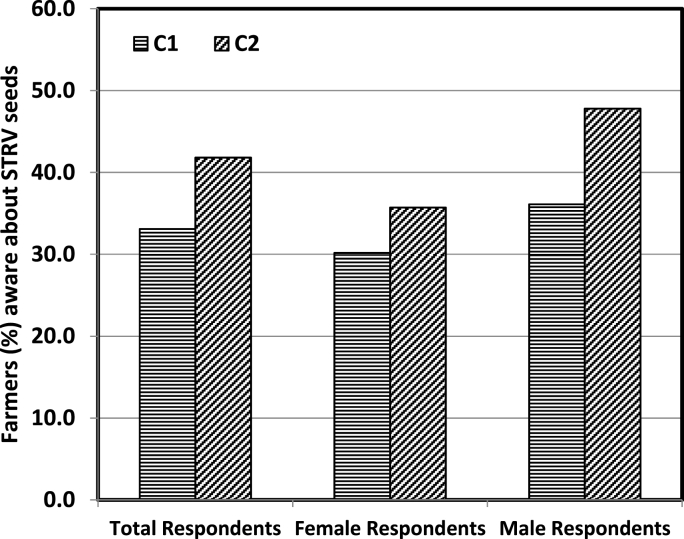
Proportion (%) of farmers aware about STRV seeds with local sources (other farmers and relatives) as the basis of their information.

Regarding the sources of information, nearly 33% of the respondents in C_1_ learnt about drought tolerant seeds from local sources like relatives and other villagers. The corresponding statistic for the C_2_ group was 42% (Fig. 8). Although, female farmers also contributed towards the difference, it's mainly driven by male farmers as 48% of the males in C_2_ learnt about new seeds from non-NGO local sources compared to the 36% in group C_1_. The local sources of information can serve as an effective and practically potential means to disseminate the technology in a sustainable manner [35].

**Fig. 9:**
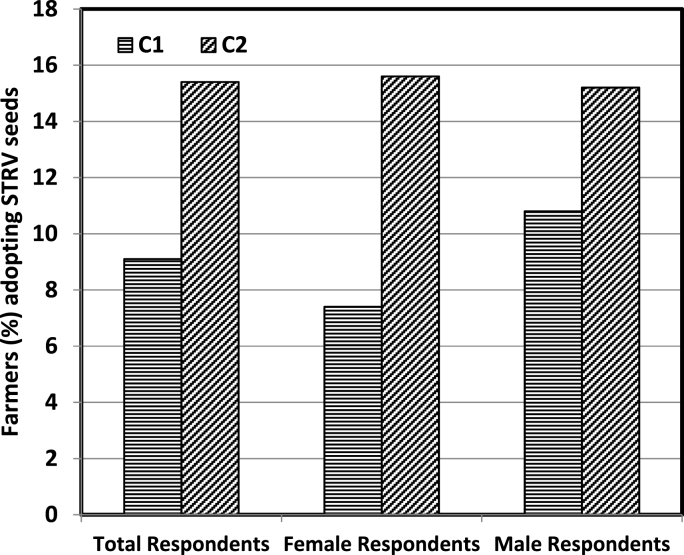
Impact of informational intervention on adoption of STRV seeds.

Similarly, 9% of the farmers in C_1_ reported to have adopted the new seeds as opposed to 15% of the farmers in C_2_ group (Fig. 9). The differential adoption was predominantly driven by female farmers as 7% of these in C_1_ have adopted new seeds compared to 16% in C_2_. It is clear from the results that the information intervention was quite economical in enhancing the awareness and adoption of STRV seeds amongst the farmers over gender, but the effect was predominantly driven by female farmers. The information intervention has potentially positive impact on the perception of the farmers towards varietal replacement [36]. Providing information to end users of the technology can be a better achiever in terms of its economic feasibility compared to imparting trainings which is relatively a more expensive venture [37].

### 3.4 Impact of agro-based goods

**Fig. 10:**
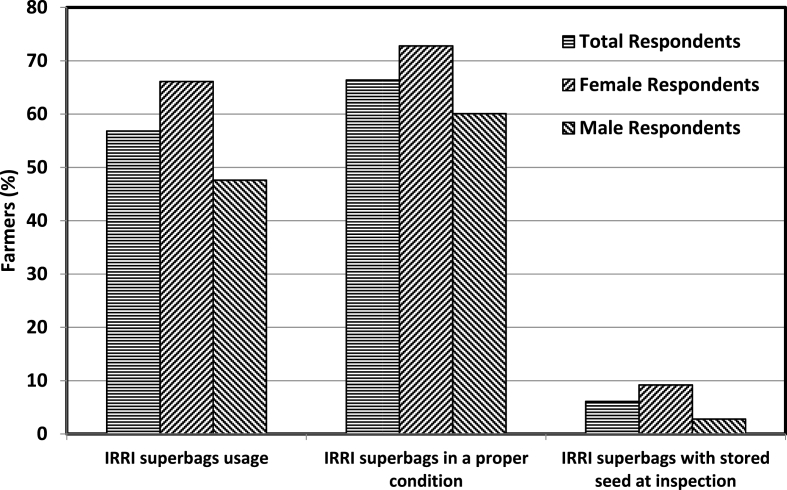
Impact of agro-based goods in terms of the proportion of farmers using IRRI super bags (only T_1_ farmers were included).

Usage of IRRI super bags was used as a benchmark to appraise the impact of agro-based goods. These bags were provided for seed storage to farmers during QSP trainings and were thus available with T_1_ farmers only. During the follow-up survey, the farmers (T_1_) were asked about their usage of IRRI super bags and about 57% of the farmers reported of using these bags during the previous year (Fig. 10). While nearly two-third (66%) of the females reported having used super bags, the corresponding share for males was around 48%, revealing that the females performed better over the males. Upon physical verification of the bags, it was observed that about 66% of the respondents have kept the super bags safely in a proper condition. Nearly three-fourth (73%) of the female farmers has safely stored the super bags, whereas nearly 60% of male respondents have accomplished so. Upon inspection, whether there was actually any seed stored in the bag, it was observed that nearly 6% of the farmers were found to have seeds stored in super bags. Moreover, almost 9% of the super bags amongst the female farmers were found to be storing seeds, while the share for male farmers was only about 3%. The less percentage of farmers irrespective of gender having seeds in the super bags is expected in the middle of the cropping season. The differences across the gender clearly indicate that female farmers performed relatively better with respect to agro-based household goods as well as from the policy perspective. Thus, it would be more desirable to provide female farmers with such products as opposed to male farmers [38]. The female farmers deliver greater value to household goods than the male farmers.

### 3.5 Empirical implications

The regression results showing an impact of QSP training are presented in Table 2. The impact of QSP trainings on T_1_ and C_1_ groups is being illustrated in column 1 of the table. The coefficient of QSP trainings reveal that imparting such trainings increase the chances of salt cleaning of seeds by almost 30% over those who neither received seeds nor any such trainings. Moreover, the coefficient is highly significant. Even after controlling the key farmer-level characteristics as well as regional factors through block-level fixed effects, QSP trainings reveal a significant impact on farmer's practices. The gender based interaction between QSP trainings and female respondents is positive but insignificant. As the coefficients are jointly significant, it may be concluded that while QSP trainings have a significant impact on salt cleaning for both male and female farmers, there is no differential impact across the gender.

**Table 2:** Impact of QSP trainings on salt cleaning and adoption of STRV seeds.

	Salt cleaning (1)	Salt cleaning (2)	STRV adoption (3)	STRV sown (4)	STRV sharing (5)
QSP Trainings	0.2873*** (0.0723)	0.2181*** (0.0619)	0.0333 (0.0746)	0.1090 (0.0784)	−0.0352 (0.0521)
QSP Trainings x Female	0.0354 (0.0787)	0.0423 (0.0691)	0.1385* (0.0796)	0.0756 (0.0913)	0.0586 (0.0610)
Female	−0.0157 (0.0373)	0.0087 (0.0331)	0.0042 (0.0576)	−0.1020* (0.0609)	0.0123 (0.0511)
Observations	434	439	439	422	439
R2	0.31	0.45	0.43	0.76	0.36
Joint significance, Female	Yes	Yes	Yes	Yes	No
Controls	Yes	Yes	Yes	Yes	Yes
Dependent variable mean	0.19	0.22	0.58	16.14	0.15

Standard error in parentheses (clustered at the village level). The dependent variable in columns 1, 2, 3 and 5 are binary in nature, equalling 1 if the respondent performed salt cleaning/adopted or shared STRV seeds. In column 4, the dependent variable is the logarithm of amount of STRV seeds sown (in Kilograms). The unit of observation is the farmer respondent. Estimation controls for household size, caste background, baseline amount of the seeds, level of land ownership, estimated rice loss in past year and BPL card ownership. Block-level fixed effects are also included.

Columns 2–5 of Table 2 compare the impact of QSP trainings across the seed recipients (T_1_ and T_2_ farmers). The results from column 2 illustrate that only QSP trainings and not seed provision nudges the farmers to use salt solution for seed cleaning. The T_1_ group which received both seeds and training were around 20% more likely to perform salt cleaning of seeds compared to T_2_ group which received seeds but no trainings. Though there is no differential impact across male and female respondents, statistically significant impact of QSP trainings for both the genders have been observed. Column 3 of the table shows the impact of QSP trainings on adoption of new seeds along an extensive margin. On the extensive margin, the impact of QSP training on adoption was higher for female farmers than for male farmers. Female farmers receiving QSP training has around 13% higher likelihood of adopting new seeds than male farmers. The impact of QSP training on the adoption seeds is not significant for male farmers, but the sum of coefficients of QSP trainings and its interaction with gender is jointly significant. This implies that QSP trainings increased an adoption of new seeds for female farmers only. The net effect being that there is nearly 17% higher likelihood of adopting STRV seeds amongst female farmers receiving QSP training over the farmers in group T_2_.

The impact of QSP trainings along the intensive margin was evaluated as natural logarithm of the quantity (in kilograms) of drought tolerant seeds sown during the follow-up year. The results in column 4 of Table 2 illustrates that there is no impact of QSP training on the amount of seeds sown for male respondents across the T_1_ and T_2_ farmers. However, there is a significant impact on female farmers. Though there is no differential impact across male and female farmers along the extensive margin, female respondents receiving QSP trainings show nearly 18% more adoption for new seeds over the farmers in T_2_ group. Thus, along both intensive and the extensive margins, the QSP training had a positive and significant impact for female farmers over the respondents who received seeds but no training. Moreover, from column 5, there is no evidence of QSP trainings impacting seed sharing. The coefficient regarding an interaction of female farmers and QSP trainings is positive but fails to attain a statistical significance. The joint sum of interaction term and QSP trainings is also non-significant, indicating that QSP training has no impact on seed sharing.

Table 3 presents the impact of information intervention on respondents who did not receive seeds. Thus, the comparison has been made between C_1_ and C_2_ farmers. Column 1 of Table 3 indicates that the information intervention increased the likelihood of knowing about STRV seeds by 10%. In column 2, it is clear that the information intervention significantly increases the likelihood of farmers towards knowing about the new seeds from local village sources such as friends, neighbours and acquaintances. In both columns 1 and 2, the positive impact of information intervention is restricted only to males and there is no corresponding impact on female farmers. In column 3, we compare the impact of information intervention on the rates of adoption of new seeds amongst non-seed recipients. The only impact of the information intervention is on females with the sum of coefficients being jointly significant. The information intervention increases the likelihood of adoption of new seeds amongst female farmers by almost 10% and there is no corresponding impact on male farmers. Therefore, from Table 3, it is clear that the information intervention helped in enhancing the knowledge of seeds amongst male farmers and also encouraged the female farmers to adopt these seeds. This implies that even minor incentives and interventions can be very effective for technological dissemination if targeted to potential receivers. This is in contrary to the implications of QSP trainings showing muted impact on seed sharing.

**Table 3:** Impact of information intervention on knowledge and adoption of STRV seeds.

	STRV knowledge (1)	STRV knowledge local sources (2)	STRV adoption (3)
Information	0.0922 (0.0560)	0.1374** (0.0692)	0.0370 (0.0451)
Information x Female	−0.0153 (0.0772)	−0.0549 (0.0859)	0.531 (0.0662)
Female	−0.0282 (0.0508)	−0.0655 (0.0593)	−0.0407 (0.0451)
Observations	502	502	502
R2	0.41	0.32	0.23
Joint significance, Female	No	No	Yes
Controls	Yes	Yes	Yes
Dependent variable mean	0.64	0.36	0.13

Standard error in parentheses (clustered at the village level). The dependent variable in each column is binary in nature, equalling 1 if the respondent knows/adopted STRV seeds. The unit of observation is the farmer respondent. Estimation controls for household size, caste background, level of land ownership, estimated rice loss in past year and BPL card ownership. Block-level fixed effects are also included.

The usage of IRRI super bags across the males and female farmers was also evaluated (Table 4). As the super bags were provided to T_1_ farmers only, therefore within the group comparisons were made across the gender to evaluate the results. Three way approach was used for evaluating the usage of IRRI super bags. First is a self-reported measure whereby the respondents informed about usage of the IRRI super bags. In the second measure, verification of the super bags was carried out to ensure whether these have been kept safely by the farmers. Third measure pertains to the physical verification to confirm if the IRRI super bags were at the moment being used to store seeds. Table 4 implies that the females have a significantly higher likelihood of self-reported usage of IRRI super bags (column 1) as over 13% more female farmers reported having used the super bags for seed storage relative to male respondents. Moreover, upon physical inspection, it was found that 10% more female respondents were presently storing seeds in the IRRI super bags (column 3) than male farmers. The results are consistent with the studies of Mehra and Hill Rojas [39] showing that females have a higher valuation for household goods and exhibit a higher propensity to use them.

**Table 4:** Impact of IRRI super bag usage by farmers.

	Super bag use self-reported (1)	Super bag safely kept (2)	Super bag with seed stored (3)
Female	0.1303** (0.0635)	−0.0426 (0.0701)	0.1010** (0.0385)
Observations	198	198	198
R2	0.52	0.51	0.25
Controls	Yes	Yes	Yes
Dependent variable mean	0.57	0.61	0.07

Standard error in parentheses (clustered at the village level). The dependent variable in each column is binary in nature, equalling 1 if the respondent replied in the affirmative way regarding super bag usage. The unit of observation is the farmer respondent. Estimation controls for household size, caste background, level of land ownership, estimated rice loss in past year, baseline amount of seeds provided and BPL card ownership. Block-level fixed effects are also included.

## 4 Conclusions

The present study provides with evidence that an increase in salt solution usage to clean seeds during the follow-up year is primarily due to the impact of QSP trainings. The trainings impacted female farmers more than the male farmers. Moreover, the impact of QSP trainings on adoption and reusability of drought tolerant seeds was more pronounced on females than on male farmers. Therefore, investment in agricultural trainings is necessary to meet the food requirements of ever-increasing population, improving food security in developing economies and enable the farmers to adapt towards changing climatic conditions. Although, agricultural trainings have substantially positive consequences across the gender of farmers, the need for women involvement in agricultural training programs is crucial to ensure that the full potential of the trainings is exploited towards enhancing the agricultural productivity. Ensuring equal opportunities for men and women in extension trainings can be helpful in increasing food production for the family and community as a whole. Therefore, the factors that prevent women from participating in training programs should be taken into consideration while planning for any such activities. Similarly, the distribution of seeds substantially enhanced its adoption and reusability amongst farmers irrespective of the gender. Nevertheless, improved seed is the primary input for modern agriculture and its distribution has direct impact on agricultural productivity, food security and sustainable economic growth. Likewise, the information intervention was quite economical in enhancing the awareness and adoption of new seeds amongst the farmers across the gender, but the effect was predominantly driven by female farmers. The informational interventions have potential to enhance the knowledge and confidence levels of both male and female farmers, thereby improving their decision making. The information intervention has potentially positive impact on the perception of farmers towards varietal replacement. Providing information to end users of the technology can be a better achiever in terms of its economic feasibility compared to imparting trainings which is relatively a more expensive venture. Furthermore, the female farmers performed relatively better with respect to agro-based household goods. It would be more desirable to provide female farmers with such products as opposed to male farmers. To summarise, incentivising farmers in general and female farmers in particular has the potential impact to boost rural economy and enhance the food security.
